# Draft Genome Sequences of *Bacillus* sp. Strains RM1(2019), RM2(2019), RM9(2019), RM11(2019), and RM15(2019), Isolated from Temperate Soils in the Hudson River Valley of New York

**DOI:** 10.1128/MRA.01230-19

**Published:** 2019-12-12

**Authors:** Rachael A. Mendoza, Swapan S. Jain, Gabriel G. Perron

**Affiliations:** a Department of Biology, Reem-Kayden Center for Science and Computation, Bard College, Annandale-on-Hudson, New York, USA; b Department of Chemistry & Biochemistry, Reem-Kayden Center for Science and Computation, Bard College, Annandale-on-Hudson, New York, USA; c Center for the Study of Land, Air, and Water, Bard College, Annandale-on-Hudson, New York, USA; Queens College

## Abstract

We report the draft genome sequences of five novel Bacillus strains isolated from temperate soils in Annandale-on-Hudson, NY. Strains RM1(2019), RM9(2019), and RM15(2019) were identified as Bacillus toyonensis, while RM11(2019) was identified as Bacillus thuringiensis. The draft genome of strain RM2(2019) was unclassified and likely represents a new species.

## ANNOUNCEMENT

*Bacillus* is a genetically and metabolically diverse genus of bacteria that have been used for environmental, medicinal, and nutritional purposes (1). The characterization of novel *Bacillus* strains thus remains a priority for understanding the evolutionary history of the genus as well as for the development of novel biotechnological applications (2).

To investigate the genetic diversity of environmental *Bacillus* spp. in the Hudson River Valley in New York, we collected 1 g of soil from five different locations on the Bard College campus located in Annandale-on-Hudson, NY. The soil samples were mixed with 9 ml of sterile water and incubated at 42°C in a shaking incubator for 1 hour (3). Following incubation at room temperature for 2 h, we plated the samples on 0.1% tryptic soy broth (TSB) agar for 24 to 48 h at 30°C (3). For each sample, we selected one white irregularly shaped colony and confirmed that the colonies were Gram positive via Gram straining. We then extracted genomic DNA using the DNeasy UltraClean microbial kit (Qiagen, Hilden, Germany). After confirming the genus identification of strains RM1(2019), RM2(2019), RM9(2019), RM11(2019), and RM15(2019) (Table 1) via 16S rRNA sequencing using the universal primers 27F (AGAGTTTGATCMTGGCTCAG) and 1492R (CCAGACTCCTACGGGAGGCAGC), whole-genome sequencing was conducted at the Microbial Genome Sequencing Center, LLC (Pittsburgh, PA) using the 151-bp paired-end read libraries designed for the Illumina MiSeq platform. We obtained a total of 1,933,616 pairs of raw reads for RM1(2019), 1,767,769 pairs of raw reads for RM(2019), 1,998,924 pairs of raw reads for RM9(2019), 2,786,858 pairs of raw reads for RM11(2019), and 3,148,200 pairs of raw reads for RM15(2019).

**TABLE 1:** Summary of the draft genome sequences for environmental *Bacillus* sp. strains isolated from soil in Annandale-on-Hudson, NY, and described in this study

Isolate	Location of isolation (GPS coordinates)^*a*^	Closest species match (DDH %)	No. of contigs	Genome size (bp)	G+C content (%)	*N*_50_ (bp)	Median coverage (fold)	GenBank accession no./SRA accession no.
RM1(2019)	42°1′12″N, 73°54′28″W	Bacillus toyonensis NCIMB 14858 (98.7)	52	5,820,521	34.94	308,271	77	WBOR00000000/SRR10120510
RM2(2019)	42°1′49″N, 73°54′17″W	Bacillus thuringiensis ATCC 10792 (69.5)	80	5,968,737	34.80	239,180	68	WBOQ00000000/SRR10120509
RM9(2019)	42°1′47″N, 73°54′16″W	Bacillus toyonensis NCIMB 14858 (94.8)	38	5,627,226	35.11	719,831	73	WBOP00000000/SRR10120508
RM11(2019)	42°1′12″N, 73°54′28″W	Bacillus thuringiensis ATCC 10792 (81.7)	65	5,928,652	34.79	326,972	107	WBOO00000000/SRR10120507
RM15(2019)	42°1′26″N, 73°54′23″W	Bacillus toyonensis NCIMB 14858 (94.6)	56	5,819,922	34.98	390,194	125	WBON00000000/SRR10120506

a GPS, global positioning system.

We assembled the genomes using the bioinformatic pipeline described by Shrestha et al. (4). Briefly, raw reads were trimmed using Trimmomatic v0.36 with the following parameters: SLIDINGWINDOW, 4:15; LEADING, 3; TRAILING, 3; MINLEN length, 50 (5). The trimmed reads were then assembled using SPAdes *de novo* v3.11 testing assemblies, with k-mer lengths of 21, 33, 55, 77, 99, and 127 (6). Contigs smaller than 500 bp or with low coverage (<2-fold) were filtered out. Assembly statistics were estimated using QUAST v4.5 and BBMap, and the species identity was estimated for each strain using the Genome BLAST Distance Phylogeny approach (7), as implemented on the Type (Strain) Genome Server (TYGS), using default settings (8).

We found that the draft genomes of strains RM1(2019), RM9(2019), and RM15(2019) were most closely related to that of Bacillus toyonensis NCIMB 14858 (NCBI assembly number ASM49628v1), a nonpathogenic strain that was first isolated in Japan as strain BCT-7112 (9), with DNA-DNA hybridization (DDH) values of 98.7%, 94.8%, and 94.6%, respectively. The three draft genomes ranged in size from 5,627,226 bp to 5,820,521 bp, with an average G+C content of 35.01% and an average median coverage of 92-fold. We found that the draft genome of strain RM11(2019) was most closely related to that of Bacillus thuringiensis ATCC 10792 (NCBI assembly number ASM211944v1), with a G+C content of 34.74% and a median coverage of 107-fold. Finally, we found that the draft genome of strain RM2(2019) was most closely matched to those of B. thuringiensis ATCC 10792 and B. cereus strain ATCC 14579 (NBCI assembly number ASM782v1), with DDH values of 69.5% and 65.9%, respectively, indicating that the classification of RM2(2019) most likely falls into a new species within the *Bacillus* genus (10). Strain RM2(2019) harbors a smaller genome than that of B. thuringiensis but larger than that of B. cereus (5,968,737 bp versus 6,326,280 bp and 5,427,083 bp, respectively) and a smaller G+C content than those of both strains (34.80% versus 34.94% and 35.31%, respectively).

### Data availability.

The draft genome and raw read sequences for each strain have been deposited in DDBJ/EMBL/GenBank under the accession numbers listed in Table 1.

## References

[1] Liu Y, Lai Q, Göker M, Meier-Kolthoff JP, Wang M, Sun Y, Wang L, Shao Z 2015 Genomic insights into the taxonomic status of the *Bacillus cereus* group. Sci Rep 5:14082. doi:10.1038/srep14082. 26373441 PMC4571650

[2] Harwood CR 1989 Introduction to the biotechnology of Bacillus, p 1–4. *In* Harwood CR (ed), Bacillus. Springer US, Boston, MA.

[3] Al-Humam NA 2016 Heat-shock technique for isolation of soil Bacillus species with potential of antibiotics secretion in Saudi Arabia. Br Microbiol Res J 17:1–6. doi:10.9734/BMRJ/2016/29126.

[4] Shrestha SD, Guttman DS, Perron GG 2017 Draft genome sequences of 10 environmental Pseudomonas aeruginosa strains isolated from soils, sediments, and waters. Genome Announc 5:e00804-17. doi:10.1128/genomeA.00804-17. 28839021 PMC5571407

[5] Bolger AM, Lohse M, Usadel B 2014 Trimmomatic: a flexible trimmer for Illumina sequence data. Bioinformatics 30:2114–2120. doi:10.1093/bioinformatics/btu170. 24695404 PMC4103590

[6] Bankevich A, Nurk S, Antipov D, Gurevich AA, Dvorkin M, Kulikov AS, Lesin VM, Nikolenko SI, Pham S, Prjibelski AD, Pyshkin AV, Sirotkin AV, Vyahhi N, Tesler G, Alekseyev MA, Pevzner PA 2012 SPAdes: a new genome assembly algorithm and its applications to single-cell sequencing. J Comput Biol 19:455–477. doi:10.1089/cmb.2012.0021. 22506599 PMC3342519

[7] Meier-Kolthoff JP, Auch AF, Klenk H-P, Göker M 2013 Genome sequence-based species delimitation with confidence intervals and improved distance functions. BMC Bioinformatics 14:60. doi:10.1186/1471-2105-14-60. 23432962 PMC3665452

[8] Meier-Kolthoff JP, Göker M 2019 TYGS is an automated high-throughput platform for state-of-the-art genome-based taxonomy. Nat Commun 10:2182. doi:10.1038/s41467-019-10210-3. 31097708 PMC6522516

[9] Jiménez G, Urdiain M, Cifuentes A, López-López A, Blanch AR, Tamames J, Kämpfer P, Kolstø A-B, Ramón D, Martínez JF, Codoñer FM, Rosselló-Móra R 2013 Description of Bacillus toyonensis sp. nov., a novel species of the Bacillus cereus group, and pairwise genome comparisons of the species of the group by means of ANI calculations. Syst Appl Microbiol 36:383–391. doi:10.1016/j.syapm.2013.04.008. 23791203

[10] Chun J, Rainey FA 2014 Integrating genomics into the taxonomy and systematics of the Bacteria and Archaea. Int J Syst Evol Microbiol 64:316–324. doi:10.1099/ijs.0.054171-0. 24505069

